# The oral-amplifier hypothesis: linking oral inflammation to intestinal microbial activity in autism spectrum disorder

**DOI:** 10.3389/fmicb.2026.1940902

**Published:** 2026-09-18

**Authors:** Yikai Chen, Shaowei Li, Hao Lin, Fangcheng Zou, Wenxin Wang

**Affiliations:** 1 Center for Precision Medicine, School of Medicine and School of Biomedical Sciences, Huaqiao University, Xiamen, Fujian, China; 2 The Children’s Hospital, Zhejiang University School of Medicine, Hangzhou, Zhejiang, China; 3 Fujian Medical University, Fuzhou, Fujian, China; 4 Beijing Key Laboratory of Intelligent Drug Research and Development for Mental Disorders; National Clinical Research Center for Mental Disorders; National Center for Mental Disorders; Beijing Anding Hospital, Capital Medical University, Beijing, China; 5 The Second Affiliated Hospital of Henan Medical University, Henan Key Laboratory of Biological Psychiatry (Henan Medical University), Xinxiang, Henan, China; 6 Brain Institute, Henan Academy of Innovations in Medical Science, Zhengzhou, Henan, China

**Keywords:** autism spectrum disorder, gingival inflammation, intestinal activity, microbial ecology, oral-gut transmission, periodontal inflammation, strain tracking

## Abstract

The oral-amplifier hypothesis proposes a testable sequence linking oral inflammation to intestinal microbial activity. Gingival or periodontal inflammation may alter the identity and quantity of strains released into saliva. Gastrointestinal conditions may then clear those strains, permit repeated passage, or support local activity. A participant-matched strain is an oral and intestinal population from the same person that meets prespecified genomic, coverage, and contamination criteria. The hypothesis predicts that greater inflammation-associated oral strain output will precede increased abundance or activity of the genomically matched intestinal strain. The lag is calibrated to the participant’s measured gastrointestinal transit time. The first proposed study tests this lagged within-person relationship, termed source-to-activity coupling. It asks whether community resistance, mucosal immunity, and local resources modify the relationship’s magnitude, delay, or persistence. The second proposed study is a controlled oral-health intervention in which reduced gingival inflammation must accompany a verified reduction in candidate-strain salivary output. It then tests whether matched intestinal activity declines after that verified source reduction. ASD is methodologically informative because oral-care needs, diet, medication exposure, gastrointestinal symptoms, immune state, and transit time may vary together within individuals. This variation provides measurable contrasts for testing oral output and gastrointestinal filtering but does not make the mechanism ASD-specific or causal in ASD. Together, the two proposed studies test whether increased oral output precedes transit-calibrated matched intestinal activity and verified source reduction produces downstream decline. Concordance would support a reversible, strain-specific oral contribution, whereas failure of either temporal pattern would constrain or refute the hypothesis.

## 1 Introduction: from association to a testable cross-niche hypothesis

Studies of the gut microbiome in autism spectrum disorder (ASD) have mainly compared fecal community composition between diagnostic groups. They have identified microbiota-related differences in some individuals, but causality remains unresolved. Microbial changes may influence gastrointestinal, immune, or neurobehavioral features. These same features may alter diet, medication use, transit, and microbial ecology. Diet and medication can rapidly reshape the gut microbiome (Yap et al., 2021; David et al., 2014). Stool consistency and population characteristics also explain substantial variation (Morton et al., 2023; Vandeputte et al., 2016; Falony et al., 2016). Dietary selectivity provides another source of variation (Esteban-Figuerola et al., 2019; Sharp et al., 2013). A diagnostic contrast therefore combines several dynamic exposures with reverse-causal pathways.

The oral cavity offers a more specific starting point. Here, an ecological source is a localized reservoir that releases a measurable, strain-resolved output into another habitat. Oral microbes enter saliva through biofilm turnover and epithelial shedding. Oral and fecal communities remain distinct in healthy adults (Segata et al., 2012). However, strain-resolved studies show microbial movement along the gastrointestinal tract and increased oral-fecal transmission in some diseases (Schmidt et al., 2019). Oral-origin organisms can also persist and affect the intestine when ecological conditions permit (Atarashi et al., 2017). The central question is whether a measured oral-source change predicts later activity of the same strain in the intestine.

ASD is methodologically informative because oral-care difficulty, gingival inflammation, food selectivity, medication exposure, gastrointestinal symptoms, immune state, and measured transit time may vary together within individuals. This co-variation provides measurable contrasts for testing oral output and gastrointestinal filtering, but it does not imply that the mechanism is ASD-specific or causes ASD. Dietary and gastrointestinal evidence supports several of these factors (Yap et al., 2021; Vandeputte et al., 2016; Esteban-Figuerola et al., 2019; Sharp et al., 2013). Oral-health studies document additional barriers and disease burden (Prynda et al., 2025; Ferrazzano et al., 2020; Bartolome-Villar et al., 2016). Access and sensory barriers are also well described (Lai et al., 2012; Stein et al., 2013). Existing ASD studies have generally profiled oral and intestinal communities separately (Manghi et al., 2024; Qiao et al., 2018; Kong et al., 2019). Other studies likewise report compartment-specific profiles (Abdulhaq et al., 2021; Ragusa et al., 2020). These comparisons describe ecological states but cannot establish the direction, magnitude, or timing of microbial input. The proposed study design instead follows a participant-specific strain from the mouth to its intestinal trajectory.

The hypothesis has four steps. First, gingival or periodontal inflammation modifies the oral reservoir. Second, the modified reservoir changes the strain-resolved output released in saliva. Third, gastric survival, transit, resident-community resistance, mucosal immunity, and local resources filter that output. Fourth, a participant-matched strain is cleared, remains dependent on repeated oral input, or becomes locally active. Source amplification is a within-person increase in oral strain output relative to baseline. Intestinal ecological realization requires arrival, persistence, and strain-attributable activity. The analysis unit is participant × strain × time, and each participant serves as the primary reference.

## 2 A stepwise and falsifiable cross-niche mechanism

Oral reservoirs provide a continuous microbial input to the gastrointestinal tract. The size of this input varies with biomass, dentition, salivary flow, biofilm area, hygiene, and swallowing. Gingival inflammation may alter it through ecological and host-mediated processes. Crevicular fluid, bleeding, tissue breakdown, heme, peptides, and oxygen gradients can change nutrient availability and biofilm structure. Candidate host modifiers include IL-1β, IL-6, IL-8, TNF-α, prostaglandin E2, neutrophil recruitment, epithelial permeability, and tissue remodeling. These factors are plausible correlates or modifiers, not proven strain-specific amplifiers (Jiang et al., 2025). Antimicrobial peptides may suppress susceptible organisms while favoring tolerant biofilm members. Evidence from caries provides an oral ecological precedent, not direct support for this hypothesis (Jiang et al., 2026). Experimental gingivitis studies show reversible changes in saliva and dental niches (Hall et al., 2023; Keijser et al., 2025). A valid test must therefore distinguish a broad community shift from increased output of a prespecified strain.

The framework uses four source terms. Effective oral strain output is the amount of a selected strain released per unit time. Source modulation is any inflammation-associated change in that output. Source amplification is a positive within-person change from baseline. Source restructuring is a change in strain composition without a uniform increase. Cross-niche amplification requires source amplification followed by a concordant, lagged increase in the matched intestinal strain. A participant-matched strain must satisfy prespecified criteria for genomic similarity, coverage, microdiversity, and contamination control. Species-level identity alone is insufficient.

Intestinal ecological realization has three measurable components: arrival, persistence beyond an interval calibrated to the participant’s measured gastrointestinal transit time, and strain-attributable activity. Strain redistribution refers to strains that change in different directions and is one form of source restructuring. The complete pathway runs from inflammation to oral output, gastrointestinal filtering, and intestinal activity. These definitions prevent a broad community change from being mislabeled as strain-specific amplification.

The hypothesis requires support for each ordered link. Inflammation must precede a within-person change in oral strain output. That change must precede recovery or activity of the matched intestinal strain within a lag calibrated to the participant’s measured gastrointestinal transit time. Prespecified gastrointestinal conditions should modify the response as predicted. Finally, verified source reduction should produce a corresponding downstream decline. A failure at any link identifies where the proposed sequence breaks.

The core relationship is:

Δ Intestinal strain activity = *f*(Δ effective oral strain output, gastrointestinal gate measures, lag calibrated to measured gastrointestinal transit time).

This expression yields three predictions. Source-to-activity coupling is a lagged within-person relationship in which a change in effective oral strain output is followed by altered abundance or activity of the participant-matched intestinal strain. The lag is calibrated to the participant’s measured gastrointestinal transit time. Gastrointestinal conditions should modify the magnitude, delay, or persistence of this relationship. Verified source reduction should be followed by a decline in matched intestinal activity. The gastrointestinal tract is therefore treated as a series of filters rather than a passive conduit. The same oral input may be cleared, repeatedly transmitted, or maintained locally. The outcome depends on survival, transit, community resistance, immunity, and resources.

Transmission studies usually ask whether the same strain appears in oral and intestinal samples. The oral-amplifier hypothesis asks whether inflammation-related oral output predicts the magnitude and timing of downstream activity. The proposed source-reduction intervention then tests whether that activity declines after reduced gingival inflammation and a verified reduction in candidate-strain salivary output. Detection alone is not treated as proof of colonization.

The cross-niche mechanism and the two proposed future empirical study designs are summarized in Figure 1.

**Figure 1:**
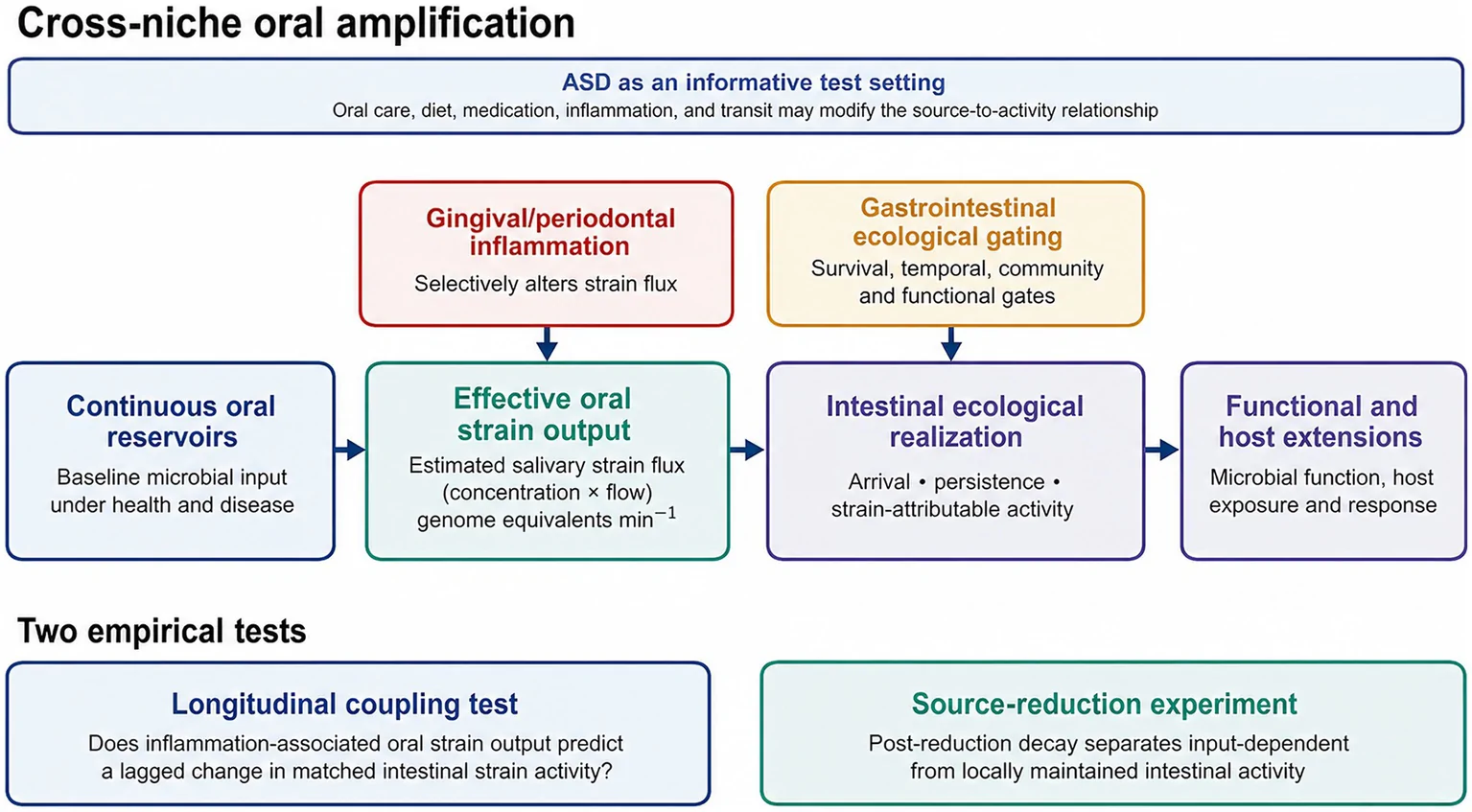
Cross-niche architecture and two proposed future empirical study designs for the oral-amplifier hypothesis. Gingival or periodontal inflammation may alter strain-resolved oral output. Gastrointestinal survival, transit, resident-community, host, and functional conditions determine whether a participant-matched strain is cleared, repeatedly detected, or remains active. The proposed longitudinal study tests whether oral output precedes a change in matched intestinal activity after a lag calibrated to measured gastrointestinal transit time. It also tests whether gastrointestinal conditions modify that relationship. The proposed source-reduction intervention tests whether verified reduction of oral inflammation and strain output produces downstream decay. The decay pattern distinguishes input-dependent activity from locally maintained intestinal activity. Functional and host outcomes are downstream extensions rather than assumed components of the core mechanism.

## 3 Three mechanistic stages

### 3.1 Measuring oral strain output

Effective oral strain output is estimated from absolute strain concentration, such as genome equivalents /mL, multiplied by salivary flow in mL/min. The proxy is reported as genome equivalents min − 1. Samples should be collected within the same clock-time window under standardized fasting, hydration, oral-hygiene, and stimulation conditions. Medication, illness, recent stress, and time since eating should be recorded. A validation subset should provide repeated within-day measurements. Sensitivity analyses should account for collection time and flow state. Output should be integrated over a prespecified interval rather than inferred from a single concentration. Unstimulated saliva is preferred for the primary analysis, with stimulated saliva analyzed separately. Absolute quantification reduces compositional bias (Barlow et al., 2020), and collection methods must remain consistent (Proctor and Carpenter, 2016; Bang et al., 2023). Subgingival abundance and activity characterize the reservoir that generates the salivary output.

DNA-based genome-equivalent measurements include dead cells and extracellular DNA. Salivary strain flux must therefore be described as genomic output unless viability is measured separately. DNase pretreatment can reduce extracellular-DNA signals. Validated viability PCR can reduce signals from membrane-compromised cells. Key strains should also be assessed with RNA:DNA ratios, targeted transcription, culture, or another activity-sensitive assay where feasible. Concordant genomic and activity signals support biologically effective input. A DNA-only signal indicates exposure to genetic material, not viable propagation.

Each participant’s repeated baseline is the primary reference. Between-person comparisons remain useful, but similar inflammation scores may accompany different reservoirs, salivary flow rates, plaque areas, and shedding rates. Within-person deviations isolate changes associated with inflammation while controlling stable personal characteristics. The main exposure is therefore the within-person change in estimated salivary strain flux.

Candidate strains must be selected before intestinal outcomes are examined. Selection requires reproducible oral detection, adequate genomic coverage, measurable output, and a within-person association with oral inflammation. The rule may use oral and clinical data, an independent discovery set, or prespecified external evidence. It must not use the intestinal outcome under test. This separation prevents circular selection of strains with the strongest oral-stool association.

Inflammation can produce three source patterns. Amplification increases the effective input of a strain. Suppression decreases it. Restructuring changes the distribution of strains without a uniform increase. Each pattern generates a different intestinal prediction. Amplified strains should increase downstream when gastrointestinal conditions permit. Suppressed strains may decline, while restructuring may change which oral strains become active in the intestine.

### 3.2 Gastrointestinal ecological gating

Gastrointestinal ecological gating refers to measurable conditions that influence successive steps after swallowing. Survival gates include gastric acid, proton-pump inhibitor exposure, bile, food-matrix protection, and innate antimicrobial activity. Temporal gates include transit, residence time, constipation, and stool consistency. Community gates determine whether resident organisms exclude, transiently accommodate, or maintain incoming strains. Transmission and transit studies support these processes (Vandeputte et al., 2016; Schmidt et al., 2019; Atarashi et al., 2017). Inflammation and medication provide further evidence (Imhann et al., 2016; Kitamoto et al., 2020). Host gates include barrier integrity, mucus, secretory IgA, antimicrobial peptides, and inflammatory signaling. A review of barrier modulation in metabolic disease provides a cross-disease precedent (Su et al., 2023), not evidence for the same mechanism in ASD. TLR4-NF-κB signaling can alter cytokines and barrier conditions during intestinal inflammation. Evidence from inflammatory bowel disease is a mechanistic precedent, not evidence for the same pathway in ASD (Jiang et al., 2025). Functional gates determine whether an arriving strain expresses an intestinally adapted program. All candidate gates must be defined before matched-strain outcomes are examined.

The gates should be modeled separately because they act at different transitions and may interact. The primary model should include a small, preregistered set of interactions supported by a causal diagram. Examples include oral output × acid suppression, oral output × transit time, and oral output × baseline niche occupancy. Penalized, Bayesian, or machine-learning models may support secondary exploration. Such models require out-of-sample validation and interpretable effect displays. Acid suppression may improve survival, slower transit may shift the lag, and resident communities may prevent maintenance. Mucosal immunity may further alter establishment or activity. This staged strategy avoids an unstable model that attempts to estimate every interaction at once.

Gastrointestinal gates should create heterogeneity in source-to-activity coupling. Strong survival barriers and rapid transit favor attenuation or brief input-dependent detection. Longer residence may increase and delay recovery. Reduced community resistance or favorable local conditions may permit sustained intestinal activity that partly decouples from oral output. The gates therefore determine whether oral input is attenuated, repeatedly transmitted, or maintained locally.

### 3.3 Intestinal ecological realization

Intestinal ecological realization occurs when an incoming oral strain progresses from detection to persistent, strain-attributable activity. Arrival requires prospective recovery of a participant-matched strain from the same person. The match must satisfy assay-specific criteria for genomic similarity, microdiversity, coverage, and negative controls (Olm et al., 2021). Cross-participant matches, species-level resemblance, and low-coverage calls do not qualify. Only oral strains with adequate and reproducible coverage enter the downstream analysis.

Persistence requires quantitative continuity across serial intestinal samples. One candidate criterion is detection above the assay-specific quantification limit in three consecutive evaluable stool samples. These samples should span more than one measured transit interval. The criterion is illustrative and must be calibrated in a pilot study. Sensitivity analyses should vary both the number of detections and the interval length. Absolute load and uncertainty intervals help distinguish trace arrival from a sustained trajectory. Technically inadequate samples are non-evaluable, not negative.

Functional realization requires activity attributable to the participant-matched strain. Metatranscriptomics can identify expressed genes and pathways, while replication metrics may indicate growth (Franzosa et al., 2014; Korem et al., 2015). Candidate pathways may produce p-cresol, 4-ethylphenyl sulfate (4-EPS), short-chain fatty acids, tryptophan metabolites, bile acids, or sphingolipids. Taxon presence alone does not establish production. Gene content or expression should therefore be linked to metabolomics in stool, plasma, or urine. Multi-compartment metabolomics provides one useful workflow (Cheng et al., 2022). The *Flavonifractor plautii*-phytosphingosine study illustrates a testable species-product relationship outside ASD (Li et al., 2025). The framework does not assume one universal ASD-associated taxon. Candidate taxa must be nominated by reproducible oral amplification, participant-matched recovery, and relevant functional capacity. Input-dependent activity tracks oral output and declines after verified source reduction. Locally maintained activity persists beyond a window calibrated to measured gastrointestinal transit time.

Serial stool samples describe the downstream population trajectory. Strain-resolved transcription and replication provide evidence of activity. Selected mucosal or spatial studies can test how closely fecal trajectories represent local intestinal populations.

An intestinal sample-strain pair should enter the primary analysis only when sequencing depth and strain-specific detection probability are adequate. The assay must also meet the quantification limits required for correspondence, transcription, or replication analysis. Samples below these thresholds are non-evaluable rather than inactive. Replication metrics likewise require prespecified minimum coverage.

Repeated immigration and local activity can produce similar fecal detection patterns. Repeated immigration should track oral output closely, show a short lag, and decline rapidly after verified source reduction. It should also vary with transit and stool consistency. Local maintenance should show slower decay, partial decoupling from oral output, and continuing strain-attributable transcription or replication. A locally active population may also adopt an expression state distinct from its oral source.

Post-reduction decay is therefore the main discriminator. A decline on the scale of gastrointestinal transit supports input-dependent activity. Slower decay with continuing activity supports local maintenance, an alternative source, or a longer lag. A fast decline followed by slower persistence indicates a mixed mode. These patterns avoid forcing every strain into a simple immigration-versus-colonization category.

## 4 Two proposed future empirical study designs

These are proposed future empirical study designs and were not implemented in this review. Existing studies of oral-gut transmission, transit, strain resolution, and periodontal intervention support individual design components and feasibility. However, no cited study has tested the complete oral-inflammation-to-intestinal-activity sequence. The longitudinal study tests temporal ordering and effect modification. The intervention tests source dependence and reversibility. Together, they ask whether a participant-matched intestinal signal follows oral output, changes with gastrointestinal conditions, and declines after verified oral source reduction. The primary predictions, temporal patterns, and mechanistic interpretations are summarized in Table 1.

**Table 1:** Mechanistic links, research questions, temporal predictions, and interpretations for the two proposed future empirical study designs of the oral-amplifier hypothesis.

Mechanistic link	Primary prediction	Operational evidence	Expected temporal pattern	Mechanistic interpretation
Inflammation to oral source change	Increasing gingival or periodontal inflammation predicts a strain-specific increase, decrease, or redistribution in effective oral strain output relative to the participant’s baseline.	Absolute strain concentration × salivary flow; subgingival reservoir confirmation; viability or activity evidence where biologically effective output is claimed.	The oral-source change accompanies or follows inflammation and precedes the intestinal response.	Concordant increases support source amplification; decreases or redistribution indicate source modulation or restructuring.
Propagation through gastrointestinal gates	Greater effective oral strain output predicts greater abundance or activity of the same participant-matched intestinal strain.	Strain-resolved oral-stool matching; prospective serial recovery; strain-resolved transcription, replication, or pathway expression.	A lag calibrated to measured gastrointestinal transit time, with magnitude, delay, and persistence modified by survival, transit, resident-community, host, and functional gates.	Tight short-lag coupling supports input-dependent activity; continued activity with partial decoupling supports local maintenance.
Source reduction to downstream response	A larger verified reduction in effective oral strain output predicts a larger lagged decline in matched intestinal activity.	Controlled oral-health intervention; clinical improvement; verified reduction in oral strain output; serial matched intestinal activity.	Rapid decay near measured transit favors passage or repeated immigration; slower decay favors local maintenance, another source, or a longer lag.	Reversible source and intestinal changes support the hypothesis; persistence after verified source reduction localizes the remaining mechanism downstream.

Strain-resolved transcription and replication should be interpreted jointly with temporal continuity and post-perturbation decay. Serial stool measurements are non-invasive proxies for intestinal recovery and activity. Estimated salivary strain flux approximates input available for swallowing and is not equivalent to swallowed dose. Arrival, persistence, and functional realization must be reported separately; technically inadequate samples are classified as non-evaluable rather than negative.

### 4.1 Proposed longitudinal coupling study

This is a proposed prospective longitudinal repeated-measures observational study. The longitudinal study has one primary objective: to determine whether a within-person increase in inflammation-associated oral strain output precedes increased abundance or activity of the participant-matched intestinal strain. It addresses three research questions. The first asks whether oral inflammation precedes increased effective oral strain output. The second asks whether that increase precedes matched intestinal arrival, persistence, or activity within a lag calibrated to measured gastrointestinal transit time. The third asks whether prespecified gastrointestinal conditions modify the response magnitude, delay, or persistence. The exposure is the within-person deviation in effective oral strain output from baseline. The outcome is the lagged change in abundance or activity of the participant-matched intestinal strain. The analysis unit is participant × strain × time.

Repeated subgingival, salivary, and stool sampling provides temporal alignment. Gingival or periodontal inflammation should be measured continuously. Subgingival samples locate the source reservoir. Standardized saliva and flow measurements estimate salivary strain flux, while stool samples trace downstream recovery and activity. Diet, medication, oral hygiene, stool consistency, and transit must be recorded concurrently. These factors can alter the source, gastrointestinal gates, or sample interpretation. ASD and non-ASD participants can be sampled across the inflammation distribution instead of being divided into arbitrary high and low groups.

The proposed longitudinal study has three linked expectations. First, increased inflammation should precede increased output of prespecified inflammation-associated strains. Other strains may be suppressed or redistributed. Second, greater oral output should precede a concordant increase in arrival, persistence, or activity of the matched intestinal strain. Third, survival, temporal, community, host, and functional gates should modify the magnitude, lag, or persistence of that response.

Mixed-effects or hierarchical models can represent clustering by participant and strain. Within-person centering separates personal baselines from between-person differences. Distributed-lag models estimate the temporal response, and prespecified interaction terms test selected gastrointestinal modifiers. A future protocol must define the exact model, diagnostics, and validation plan.

Three outcome patterns organize interpretation of the longitudinal study. A lagged increase in matched intestinal activity after higher oral output supports propagation. Systematic variation in magnitude, delay, or persistence across prespecified gastrointestinal conditions supports gating. An oral source change without downstream activity localizes attenuation to a gastrointestinal gate. Intestinal activity without the prespecified source change suggests another source, earlier establishment, or an incorrect lag. Post-reduction decay is evaluated separately by the intervention in Section 4.2.

### 4.2 Proposed source-reduction intervention

This is a proposed controlled oral-health intervention study. The source-reduction intervention has one primary objective: to determine whether matched intestinal activity depends on continued oral input. It addresses three research questions. The first asks whether oral-health care reduces gingival inflammation and produces a confirmed reduction in candidate-strain salivary output. The second asks whether verified source reduction is followed by a lagged decline in matched intestinal activity. The third asks whether the decay pattern is consistent with transit-dependent input, local maintenance, or a mixed mode. The proposed intervention therefore requires three sequential observations: reduced oral inflammation, verified reduction in candidate-strain salivary output, and a lagged change in the matched intestinal strain. Reduced inflammation alone does not confirm source reduction.

The intervention may combine professional cleaning, plaque control, and treatment of inflammation. A usual-care, delayed-intervention, or stepped-wedge comparison should be included. Periodontal treatment can alter oral microbial functions over time (Simpson et al., 2024). The primary target-engagement measure is a prespecified reduction in salivary strain flux. Reduced subgingival abundance or activity provides additional evidence that the source reservoir changed. For source restructuring, target engagement is a prespecified normalization of strain allocation.

Systemic antibiotics and locally delivered antimicrobials should be excluded from the primary mechanistic intervention where feasible. If required, they should be analyzed as separate components because they may alter intestinal outcomes independently of oral output.

The main prediction is continuous rather than binary. Participants and strains with the largest confirmed reversal of oral output should show the largest lagged decline in matched intestinal activity. Concurrent controls help distinguish intervention effects from natural variation, regression to the mean, and seasonal or dietary changes.

A rapid decline on the scale of transit supports an input-dependent component. Persistence beyond that window, especially with transcription or replication, supports local maintenance. A fast initial decline followed by slower persistence indicates a mixed mode. No change after verified target engagement suggests an alternative source, established local maintenance, or an incorrect strain match.

The perturbation also clarifies what has been amplified. A reversible oral increase followed by a reversible intestinal increase provides the strongest evidence for amplified input. Source restructuring followed by a corresponding downstream shift supports redistribution. A discordant downstream response instead suggests selection within the gastrointestinal tract. The intervention is therefore a mechanistic discriminator, not only a treatment evaluation.

The longitudinal study tests temporal ordering and effect modification. The source-reduction intervention tests source dependence and reversibility. Neither design has been conducted in this article. Together, they evaluate the complete inflammation-to-source-to-activity sequence and provide complementary criteria for supporting, constraining, or refuting the hypothesis.

### 4.3 Practical feasibility and design safeguards

Testing should proceed in two stages. A pilot study should estimate recruitment, retention, salivary-flow variation, evaluable-strain yield, sequencing depth, contamination, transit time, and within-person outcome variance. These estimates should inform simulation-based power calculations for the planned hierarchical model. A confirmatory study should then lock candidate-strain rules, lag windows, persistence criteria, gate interactions, and missing-data procedures before analysis.

One illustrative schedule uses two baseline collections per week for 2 weeks, followed by weekly paired subgingival, saliva, and stool samples for 8–12 weeks. Sampling should be denser around source reduction. Follow-up must extend beyond several measured transit intervals. The pilot study should determine the final frequency needed to estimate lags without imposing unnecessary burden.

Low-biomass and strain-resolution challenges require extraction blanks, field blanks, positive controls, randomized processing, spike-in standards, and replicate confirmation near detection limits. Breadth and depth thresholds must be defined in advance. Samples that fail technical criteria are non-evaluable rather than negative. Candidate strains must be selected from oral data independently of intestinal outcomes. Key matches should be confirmed by a second method where possible.

Feasible ASD research requires individualized assent and consent, caregiver partnership, sensory accommodations, flexible home sampling, and avoidance of unnecessary invasive procedures. Professional cleaning and plaque-control support are preferred over systemic antibiotics for the proposed intervention study. Diet, medication, oral hygiene, dental treatment, acute illness, stool consistency, and transit should be recorded as time-varying covariates. Major changes should trigger prespecified exclusion windows or sensitivity analyses.

## 5 Why autism spectrum disorder is methodologically informative for testing the hypothesis

Here, informative means methodologically informative. In ASD, oral-care needs, diet, medication exposure, gastrointestinal symptoms, immune state, and measured transit time may co-vary within individuals. This variation supports tests of oral output and gastrointestinal filtering, but it does not imply that the mechanism is ASD-specific or causes ASD. Oral-care barriers may sustain gingival inflammation or complicate source reduction (Prynda et al., 2025; Ferrazzano et al., 2020; Bartolome-Villar et al., 2016). Access and sensory barriers add further variation (Lai et al., 2012; Stein et al., 2013). Food selectivity changes dietary substrates (Yap et al., 2021; Esteban-Figuerola et al., 2019; Sharp et al., 2013; David et al., 2014). Medication may alter oral ecology or gastrointestinal gates (Coury et al., 2012; Bahr et al., 2015). Constipation may prolong transit (Vandeputte et al., 2016). These exposures and developmental outcomes can be measured together in a clinically important population. Comparative cohorts remain necessary.

ASD must not be treated as a uniform biological category. Prespecified modifiers should include oral-care support needs, gingival inflammation, gastrointestinal symptoms, constipation, age, developmental stage, dietary selectivity, and medication class. Relevant co-occurring conditions include epilepsy, anxiety, attention-deficit/hyperactivity disorder, and sleep disturbance. Genetic or syndromic subtypes should be examined only when adequately powered. Immune phenotypes should be measured directly rather than inferred from an ASD diagnosis. Candidate measures include inflammatory markers, barrier markers, secretory IgA, and antimicrobial peptides. Recruitment should span the major exposure profiles. Models should use continuous modifiers with partial pooling rather than numerous underpowered subgroup tests.

The primary estimand is within-person propagation from inflammation-related salivary strain flux to lagged intestinal activity of the matched strain. ASD status and measured ASD-related exposures are prespecified modifiers of the source and gastrointestinal gates. Any ASD-related difference must be reported on a defined interaction scale with a prespecified link function and repeated-measures structure.

ASD is neither a microbial exposure nor a uniform biological category. It is a heterogeneous setting in which source and gastrointestinal modifiers may align. Stronger coupling may mean a steeper source-to-activity slope, a greater probability of persistence, a longer duration of activity, or a lower oral-output threshold. These quantities must be reported separately. Behavioral and developmental outcomes remain downstream hypotheses.

### 5.1 Bidirectional microbiota-gut-brain context

The microbiota-gut-brain axis is bidirectional. Intestinal microbial products may signal through immune, endocrine, metabolic, vagal, and barrier-related routes. Stress, behavior, sleep, diet, medication, toileting, and gastrointestinal motility may also reshape oral and intestinal ecology. Fractional-order modeling in ASD shows how delayed recovery, inflammatory thresholds, and history dependence can be represented (Qahiti et al., 2026). Here, that work motivates lagged and history-dependent analyses. It does not replace strain-resolved longitudinal evidence.

Neurobehavioral outcomes should be tested after ecological realization has been established. A downstream analysis would first link matched-strain activity to a strain-associated metabolite or immune signal. It would then test whether that signal precedes an independently measured neurobehavioral change. Reverse pathways from behavior to diet, oral care, medication, and transit must be modeled as well. This separation prevents the ecological hypothesis from becoming a claim that oral-gut transmission causes ASD.

## 6 Discussion

The oral-amplifier hypothesis shifts attention from static differences between body sites to a temporally ordered process. It links inflammation-associated oral output, gastrointestinal filtering, and persistent activity of the same strain. Microbial functions and host responses are evaluated only after this source-to-activity sequence is supported. In ASD, this framework may be particularly useful for examining within-group heterogeneity. Oral-care difficulty, gingival inflammation, dietary selectivity, medication exposure, gastrointestinal symptoms, and differences in transit time, including constipation-associated slowing, may influence different stages in different combinations across individuals.

The framework depends on the quality of its proxies. Salivary strain flux approximates the input available for swallowing. Serial stool samples approximate intestinal trajectories. Strain-resolved activity measures also require adequate abundance and sequencing depth. These limits should define sample evaluability rather than be interpreted as biological absence.

Functional and host effects lie downstream of the ecological mechanism. Candidate products include p-cresol, 4-EPS, short-chain fatty acids, indole derivatives, secondary bile acids, and sphingolipid-related metabolites. Their effects depend on pathway, dose, timing, and host context. Urinary p-cresol is elevated in small ASD cohorts (Altieri et al., 2011; Gabriele et al., 2014). Experimental p-cresol exposure alters behavior and gut ecology in mice (Bermudez-Martin et al., 2021). Experimental work links 4-EPS exposure to neurobehavioral outcomes and developmental timing (Hsiao et al., 2013; Needham et al., 2022). Short-chain fatty acids cannot be labeled uniformly beneficial or harmful without specifying molecule, concentration, site, and developmental context (Zhang et al., 2025). A source-to-function claim requires matched-strain gene content or expression, concordant metabolite measurements, temporal ordering, and a host-response endpoint. Host inflammation may also change substrates, barrier conditions, and microbial gene expression. These effects must be measured rather than assumed. The clinical relevance of this downstream pathway to ASD would require a further temporal link between strain-associated metabolic or host-response changes and independently measured clinical outcomes. Relevant outcomes could include gastrointestinal symptom burden and dimensional neurobehavioral features such as irritability, anxiety-related symptoms, sleep disturbance, feeding difficulty, or adaptive functioning. Studies in Alzheimer-model mice, metabolic constitution, and ischemic stroke provide methodological examples, not ASD-specific validation (Cheng et al., 2022; Li et al., 2025; Zhang et al., 2025).

Testing should be staged. A feasibility phase should establish recruitment, tolerability, analyte stability, contamination control, and strain recovery. A longitudinal oral-saliva-stool study should then test temporal ordering and estimate variance. A controlled oral-health intervention should test whether verified source reduction produces downstream decay. Sample size should be estimated by simulation using pilot data on within-person variance, evaluable-strain yield, autocorrelation, dropout, and plausible effect size. No universal sample size is defensible before these quantities are known. Home collection, caregiver support, sensory accommodations, and delayed-intervention or stepped-wedge designs may improve feasibility. Diet, medication, dental procedures, acute infection, oral hygiene, stool consistency, and transit must be recorded and addressed in prespecified analyses.

Clinical translation should begin with indicated, low-risk oral-health care rather than microbe-directed treatment. Relevant measures include prevention and treatment of gingival disease, accessible dental services, and individualized oral-hygiene plans. Oral strain output and matched intestinal activity remain research biomarkers. This hypothesis alone does not justify probiotics, prebiotics, antimicrobial peptides, herbal products, or targeted antimicrobials for ASD. Antimicrobial-peptide research illustrates ecology-aware oral intervention design (Jiang et al., 2026). The Astragalus review illustrates host-microbiome and barrier modulation in metabolic disease (Su et al., 2023). Neither source establishes efficacy for ASD or for the proposed pathway.

Future studies could compare oral-health support, barrier- or diet-directed care, and combined strategies. Outcomes should include benefit, burden, safety, and cost. Two ASD perspective and stigma models illustrate mathematical approaches to time-varying intervention intensity and cost (Gümüş and Teklu, 2025a, 2025b). They are not biological evidence for the oral-gut pathway. Candidate research biomarkers include gingival inflammation, biologically effective salivary strain output, matched intestinal load and transcription, transit, stool consistency, targeted metabolites, and host inflammatory or barrier markers (Cheng et al., 2022). These biomarkers could subsequently be related to dimensional gastrointestinal and neurobehavioral outcomes to determine whether the ecological pathway has clinical relevance within ASD.

The framework can also be tested in inflammatory bowel disease, metabolic disease, and other neurodevelopmental or neurodegenerative conditions. The transferable design has four elements: a measured source perturbation, a trackable strain, explicit gastrointestinal conditions, and a temporally ordered downstream response. Each cohort should preserve this sequence while defining disease-specific modifiers and outcomes. Evidence from inflammatory bowel disease, metabolic constitution, and ischemic stroke is analogical rather than direct (Jiang et al., 2025; Li et al., 2025; Zhang et al., 2025). Cross-disease extension therefore requires direct testing.

The core hypothesis is not supported if oral strain output shows no reproducible temporal coupling with matched intestinal activity. It is also not supported if verified source reduction produces no downstream change within lags calibrated to measured gastrointestinal transit time. The proposed studies require adequate assay sensitivity, evaluable strains, and measurement of major gastrointestinal modifiers. Clinical relevance to ASD would require additional evidence linking this ecological sequence to downstream functional and clinical outcomes.

## 7 Conclusion

The oral-amplifier hypothesis proposes a falsifiable, temporally ordered link between oral inflammation and intestinal activity of the same strain. The proposed longitudinal study tests whether oral output precedes matched intestinal activity after a lag calibrated to measured gastrointestinal transit time. It also tests whether gastrointestinal conditions modify this relationship. The proposed source-reduction intervention tests whether verified oral-source reduction produces downstream decay. Concordant results would support a reversible oral contribution, whereas temporal uncoupling or absent decay would constrain or refute the hypothesis. The framework does not imply that microbial change is necessary or sufficient to cause ASD.

## Data Availability

The raw data supporting the conclusions of this article will be made available by the authors, without undue reservation.
